# Autologous vs. implant-based breast reconstruction after skin- and nipple-sparing mastectomy—A deeper insight considering surgical and patient-reported outcomes

**DOI:** 10.3389/fsurg.2022.903734

**Published:** 2022-09-05

**Authors:** Maxi von Glinski, Nikla Holler, Sherko Kümmel, Mattea Reinisch, Christoph Wallner, Johannes Maximilian Wagner, Mehran Dadras, Alexander Sogorski, Marcus Lehnhardt, Björn Behr

**Affiliations:** ^1^ Department of Plastic and Hand Surgery, Burn Center, BG-University Hospital Bergmannsheil, Ruhr University Bochum, Bochum, Germany; ^2^ Department of Senology, Interdisciplinary Breast Cancer Center, Kliniken Essen-Mitte, Essen, Germany; ^3^ Clinic for Gynecology with Breast Center, Charité-Universitätsmedizin Berlin, Germany

**Keywords:** breast cancer, skin-sparing mastectomy, breast reconstruction, autologous breast reconstruction, implant-based breast reconstruction, outcome, quality of life

## Abstract

**Introduction:**

Autologous (ABR) and implant-based breast reconstruction (IBR) represent the most common procedures after skin- and nipple-sparing mastectomy. This cross-sectional study is a comprehensive analysis of ABR and IBR considering surgical and patient-reported outcomes.

**Patients and methods:**

Eligible patients underwent breast reconstruction (ABR and IBR) after skin- and nipple-sparing mastectomy between January 2014 and December 2020. Outcome parameters included quality of life (European Organisation for Research and Treatment of Cancer - EORTC - QLQ30, BR23, Breast-Q, CES-D), complication rates, aesthetic result, and breast sensitivity.

**Results:**

108 patients participated in the study (IBR: *n* = 72, age 48.9 ± 9.9 years; ABR: *n* = 36, age: 46.6 ± 7.3 years). Mean follow-up was 27.1 ± 9.3 (IBR) and 34.9 ± 20.5 (ABR), respectively. IBR patients suffered significantly more often from major complications (30.6% vs. 8.3%; *p* = 0.01), while ABR patients underwent secondary procedures significantly more often to improve the aesthetic result (55.6% vs. 29.2%, *p* = 0.004). Unilateral reconstructions revealed superior aesthetic results in ABR (n.s.), while in bilateral reconstruction IBR tended to score higher (n.s.). Scar evaluation resulted in a better result of IBR in both categories (*p* < 0.01). Breast sensitivity was severely impaired in both groups. The Breast-Q revealed a significantly higher “patient satisfaction with breast” after ABR (*p* = 0.033), while the other QoL-tests and subscales showed no significant differences between the two procedures.

**Conclusion:**

ABR is associated with a higher patient satisfaction despite the high probability of secondary procedures to improve the aesthetic outcome, whereas IBR-patients suffer more often from major complications. Furthermore, the laterality of reconstruction should be included in the individual decision-making process.

## Introduction

Around 30%–45% of all patients with breast cancer undergo mastectomy (1), and among these, almost 13% undergo contralateral prophylactic mastectomy (1, 2). Furthermore, bilateral prophylactic mastectomy in healthy women with a genetic predisposition for breast cancer has also become a common procedure (3, 4). Hence, the number of patients requesting breast reconstruction constantly increases (1). To date, the most common approaches for breast reconstruction involve prosthetic implants (implant-based breast reconstruction, IBR) and free autologous tissue transfer (autologous breast reconstruction, ABR) with the deep inferior epigastric perforator (DIEP) and the transverse rectus abdominis musculocutaneous (MS-TRAM) being the most widely used free flaps (5, 6).

Even though the literature suggests better patient-reported outcomes (7) in ABR, IBR still presents the most common reconstruction procedure after skin/nipple-sparing mastectomy (5, 8). Thanks to the development of the Breast-Q, patient-reported outcomes in breast reconstruction have widely been explored, while the health-related quality of life (HRQoL) and symptoms of depression have been neglected (8, 9). Studies giving a comprehensive overview of further clinical and surgical outcome parameters such as complication rates, aesthetic outcome, and breast sensibility of both procedures are lacking (8, 10–12). Furthermore, most studies do not include a homogenous patient collective with regard to the type of mastectomy (6, 10), which makes the interpretation of the study results difficult (8). This study aims to provide a deeper insight into patient-reported outcomes and their HRQoL following both procedures, respectively, on one side as opposed to the clinical outcome.

## Patients and methods

We conducted a cross-sectional study including all patients who underwent ABR between January 2014 and May 2020 (*n* = 127) as well as IBR following prophylactic or therapeutic mastectomy between September 2017 and May 2020 (*n* = 208) who met inclusion criteria at the two participating study centres. ABR included DIEP, MS-TRAM, and the superficial inferior epigastric artery (SIEA) flap. The indication was made after obtaining detailed informed consent considering the current indications for each procedure [sufficient donor site tissue/body mass index (BMI) in relation to the desired breast cup size/need for contralateral reduction mammaplasty, patients’ wishes, etc.].

Eligible patients had a history of ABR or IBR after skin/nipple-sparing mastectomy, a follow-up after reconstruction of at least one year, were free of cancer or metastases, and were conversant in German. Patients with an ABR after IBR and vice versa as well as any patient undergoing reconstruction with a pedicled flap or a mixed approach (bilateral reconstruction with unilateral flap and unilateral implant or additional use of a pedicled flap) were excluded.

After giving written informed consent, patients completed four self-report questionnaires to evaluate their HRQoL [European Organisation for Research and Treatment of Cancer or EORTC QLQ-C30 (13); EORTC QLQ BR 23 (14)], satisfaction with breast reconstruction [Breast-Q (15)], and the presence of depressive symptoms (Center for Epidemiology Studies Depression Scale or CES-D) in their home environment.

Cancer treatment-related symptom severity assessed by both questionnaires was not considered. The short version of the CES-D consists of 15 items representing the major symptoms of depression. Scores of 18 or higher indicate the need for further clinical evaluation of major depression (16).

Patients’ clinical and surgical data were retrospectively obtained from the electronic medical record. These comprised patients’ age, type of mastectomy and reconstruction, indication for mastectomy, preoperative breast cup size, cancer-related treatment such as breast-conserving surgery (BCS), postmastectomy radiation therapy (PMRT) before or after reconstruction and chemotherapy, comorbidities, smoking history, total hospital length of stay (LOS), history of minor and major complications, and secondary procedures. Major complications comprised implant dislocation, infection, major capsular contracture (CC, stage III or IV), and flap-related complications such as revision due to vascular compromise, partial, and total flap loss. Minor complication comprised haematoma, minor infection, or wound healing disorder of the breast or donor site. Secondary procedures included all procedures performed to improve the aesthetic appearance such as fat grafting, revision of breast shape, scar revision of breast or donor site, and contralateral reduction mammaplasty, but excluding reconstruction of the nipple-areola complex (NAC), which was rather considered as part of the primary reconstruction.

Clinical examination and photo documentation were carried out during an appointment at outpatient clinics. Clinical examination consisted of the evaluation of breast and nipple sensitivity, indurations, CC, and donor site morbidity. The evaluation of breast sensitivity comprised temperature sensation using the Tip-Therm ® and tactile point pressure sensitivity using Semmes-Weinstein monofilaments and was tested at three different locations (above the NAC, at NAC level, and below the NAC). The Tip-Therm® consists of two materials with different conductivities for heat (steel and plastic), so upon touching the skin a temperature difference is felt. The Semmes-Weinstein monofilaments are strands of nylon that vary in thickness (threshold values used: 0.07, 0.4, 2.0, 4.0, and 300 g) and are widely used to detect the loss of protective sensation in patients with peripheral neuropathy. For a reasonable comparison of breast sensitivity in unilateral reconstruction, results of the reconstructed breast were used, in bilateral reconstruction results of the breast with the superior sensibility.

**Figure 1:**
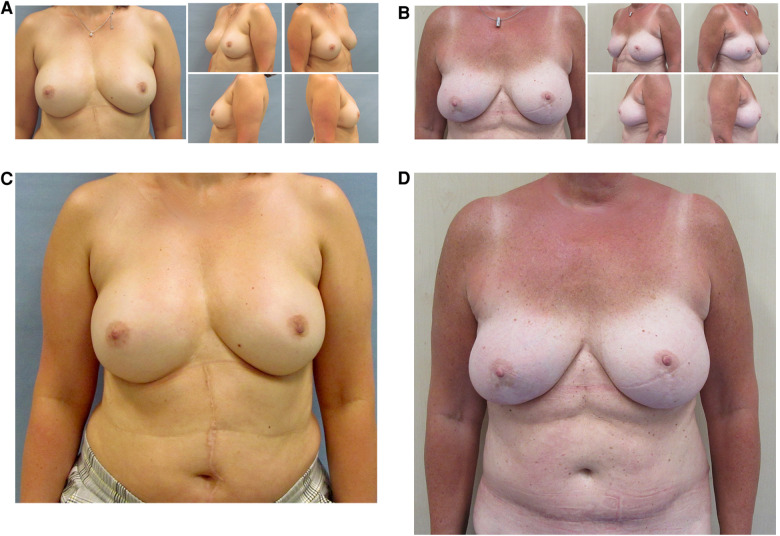
Aesthetic evaluation: pass 1 with photographs above the waistline with no hint for the performed procedure and pass 2 with appearance of the whole upper body. (**A**) 47-year-old patient with bilateral skin-sparing mastectomy and nipple-grafting after right-sided breast cancer and uncomplicated prepectoral implant-based reconstruction. Aesthetic sum score pass 1 (above the waistline): 4.60. Aesthetic sum score pass 2 (whole upper body): 5.00 (**B**) 53-year-old patient with bilateral prophylactic uncomplicated nipple-sparing mastectomy and DIEP reconstruction. Aesthetic sum score pass 1: 4.80, aesthetic sum score pass 2: 4.60. (**C**,**D**) Photographs of the whole upper body of the before-mentioned patients within aesthetic evaluation pass 2.

The aesthetic evaluation was conducted by five surgeons (two consultants, three residents) from the same department based on the photographic documentation (front view, right/left diagonal view, right/left side view) using a five-point Likert scale (1 = very unsatisfied; 5 = very satisfied). Aesthetic evaluation comprised the categories breast shape, volume, symmetry, nipple size, position, position of the inframammary fold, scars, breast–body relation, and the overall breast appearance. The evaluation was conducted in two passes. The first one showed photographs from above the waistline and, therefore, without appearance of the abdominal scar and no hint of the performed surgery. The second one showed photographs of the whole upper body asking for the overall aesthetic outcome and in ABR evaluation of the donor site scar, Figures 1A,B.

### Statistical analysis

Data were analysed using SPSS version 27 (IBM SPSS statistics, IBM Corporation, Armonk, New York, USA). Descriptive statistics and frequency distributions were generated for the sociodemographic and clinical characteristics of the sample size. Independent *t* tests were used to evaluate differences in QoL, clinical symptoms of depression, satisfaction with the breast, and clinical and aesthetic outcomes depending on the method of breast reconstruction. The chi-square test and Fisher exact test were used to determine differences in sociodemographic and clinical characteristics. A two-sided *p-*value of <0.05 was considered statistically significant.

The structure and content of the manuscript adhere to the STROBE guidelines for cohort studies.

## Results

In the study period, a total of 335 patients met the inclusion criteria of which 72 IBR patients (mean age 48.9 ± 9.9 years) and 36 ABR patients (mean age 46.6 ± 7.3 years) agreed to participate (response rate: 32.2%). 28 IBR (38.9%) and 11 ABR patients (30.6%) underwent bilateral breast reconstruction. IBR comprised 97 (97%) prepectoral and 3 (3%) subpectoral implants. ABR comprised 41 DIEP (87.2), 5 MS-TRAM (10.6%), and 1 SIEA flap (2.1%). In total, 105 (97.2%) patients had a history of breast cancer with 35 (32.4%) of them having prophylactic contralateral mastectomy, whereas 3 (2.8%) patients underwent prophylactic bilateral mastectomy. 55.3% (*n* = 26) of ABR and 37% (*n* = 37) of IBR were based on skin-sparing mastectomies with 42.6% (*n* = 20) of the NAC being reconstructed or grafted in ABR and 27 (27%) in IBR. 19.4% (*n* = 19.4) of the ABR and just 4.2% (*n* = 3) of the IBR patients had a history of BCS (*p* < 0.001). Significantly more patients with ABR had a history of PMRT before reconstruction (*p* < 0.001; 5.6% vs. 35.3%), whereas significantly more IBR patients underwent PMRT after reconstruction (*p* < 0.001; 15.3% vs. 0). Furthermore, significantly more ABR patients had a history of chemotherapy (44.4% vs. 18.1%). In total, 15 patients (13.9%) were currently smoking, 14 (12.9%) were suffering from hypertension and 1 patient (0.9%) was suffering from diabetes, without displaying a significant difference between the two groups. The mean BMI was significantly higher (*p* < 0.008) in ABR patients (26.9 ± 5.4) than in IBR patients (24.3 ± 4.3). More IBR patients had a preoperative A or B breast cup size (58.3% vs. 36.1%, n.s.), whereas more ABR patients had a preoperative C or D cup (52.8% vs. 34.5%, n.s.). Also, the mean LOS was significantly extended (*p* = 0.003) in the ABR group (median 8 vs. 4 days). All sociodemographic and clinical characteristics of the patients are summarised in Table 1.

**Table 1:** Sociodemographic and clinical characteristics of all patients who underwent primary breast reconstruction.

	IBR *n* = 72	ABR *n* = 36	*p*-value
Total count of procedures, *N* (%)	100	47	—
Prepectoral implant	97 (97)	—	
Subpectoral implant	3 (3)	—	
DIEP	—	41 (87.2)	
Ms-TRAM	—	5 (10.6)	
SIEA	—	1 (2.1)	
Mean age ± SD, year (range)	48.9 ± 9.9 (28−70)	46.6 ± 7.3 (32−69)	0.231
Laterality, *N* (%)			
Unilateral	44 (61.1)	25 (69.4)	0.154
Bilateral	28 (38.9)	11 (30.6)	
Indication, *N* (%)			
Prophylactic	1 (1.4)	2 (5.6)	0.305
Therapeutic	71 (98.7)	34 (94.4)	
contralateral prophylactic mastectomy	21 (29.6)	7 (20.6)	
NAC, *N* (%)			
Preserved (nipple-sparing ME)	63 (63)	21 (44.7)	<0.001**
Removed (skin-sparing ME)	10 (10)	6 (12.8)	
Grafted or reconstructed (skin-sparing ME)	27 (27)	20 (42.6)	
History of BCS	3 (4.2)	7 (19.4)	<0.001**
PMRT, *N* (%)^a^			
Before reconstruction	4 (5.6)	12 (35.3)	<0.001**
After reconstruction	11 (15,5)	0	
History of chemotherapy, *N* (%)	13 (18.1)	16 (44.4)	<0.001**
Currently smoking, *N* (%)	9 (12.5)	6 (16.7)	0.344
Mean BMI ± SD	24.3 ± 4.3	26.9 ± 5.4	0.008**
Breast cup size (preoperative)			0.148
A–B	42 (58.3)	13 (36.1)	
C–D	25 (34.7)	19 (52.8)	
≥E	6 (8.3)	4 (11.1)	
Comorbidities, *N* (%)			
Hypertension	8 (11.1)	6 (16.7)	0.375
Diabetes	0	1 (2.8)	0.324
Peripheral arterial disease	0	0	—
History of thrombosis	1 (1,4)	5 (13.9)	0.013*
Total hospital length of stay ± SD (range)	7.6 ± 2.7 (3−19)	9.7 ± 4.3 (6−29)	0.003**
Median	4	8	

SD, standard deviation; PMRT, Postmastectomy radiation therapy; BMI, body mass index; NAC, nipple-areola complex; BCS, breast-conserving surgery.

^a^ Count of patients with therapeutic mastectomy.

* *p* < 0.05.

** *p* < 0.01.

As shown in Table 2, mean follow-up was 27.1 (±9.3) month in the IBR group and 34.9 (±20.5) month in the ABR group (*p* < 0.045). The amount of early revision surgery within the first five days was comparable in the two groups (18.1% vs. 19.4%), whereas major complications occurred significantly more often (*p* < 0.01) after IBR (30.6% vs. 8.3%). Major complications after IBR comprised CC stage III or IV (25%), implant dislocation (8.3%), and major infection (1.4%) leading to an implant exchange once (19.4%), twice (9.7%), or more often (1.4%). Major complications in ABR comprised three cases (8.3%) of partial flap loss. In contrast, minor complications (including hematoma, minor wound healing disorder breast/donor site, minor infection) tended to occur more often after ABR (22.2% vs. 11.1%, *p* = 0.092). Furthermore, secondary procedures including fat grafting, revision of breast shape, any scar revision, and contralateral reduction mammaplasty was performed significantly more often after ABR (55.6% vs. 29.2%, *p* < 0.004).

**Table 2:** Surgical outcome parameters of all patients who underwent primary breast reconstruction.

	IBR (*n* = 72)	ABR (*n* = 36)	*p*-value
Follow-up, month (mean, SD)	27.1 ± 9.3	34.9 ± 20.5	0.045*
Revision surgery within the first 5 days, *N* (%)	13 (18.1)	7 (19.4)	0.488
Major complication, *N* (%)	22 (30.6)	3 (8.3)	0.010**
Implant dislocation	6 (8.3)	0	
Major infection	1 (1.4)	—	
Capsular contracture (stage III or IV)	18 (25.0)	—	
Implant exchange once	14 (19.4)	—	
Implant exchange twice	7 (9.7)	—	
Implant exchange ≥3	1 (1.4)	—	
Vascular compromise	—	0	
Partial flap loss	—	3 (8.3)	
Total flap loss	—	0	
Minor complications, *N* (%)	8 (11.1)	8 (22.2)	0.092
Haematoma	2 (2.8)	4 (11.1)	
Minor wound healing disorder breasts	4 (5.6)	4 (11.1)	
Minor infection	5 (6.9)	2 (5.69)	
Minor wound healing disorder of the donor site	—	1 (2.8)	
Secondary procedures, *N* (%)	21 (29.2)	20 (55.6)	0.004**
Fat grafting	0	8 (22.2)	
Other revision of breast shape	9 (12.5)	6 (16.7)	
Scar revision breast	3 (4.2)	8 (22.2)	
Dog ear resection/scar revision abdomen	—	8 (22.2)	
Contralateral reduction mammaplasty (% of unilat.)	7 (15.9)	8 (32.0)	
Temperature sensation			
Above NAC	21 (29.2)	14 (38.9)	0.269
Below NAC	18 (25.0)	9 (25.0)	0.505
Pinch tip sensation (Semmes-Weinstein)^a,b^			
Above NAC			0.532
No or 300	32 (44.4)	16 (44.4)	
4.0–0.07	40 (55.6)	20 (55.6)	
Below NAC			0.153
No or 300	46 (63.9)	18 (50.0)	
4.0–0.07	26 (36.1)	18 (50.0)	

SD, standard deviation; NAC, nipple-areola complex.

^a^ In unilateral reconstruction measured on the reconstructed site, in bilateral reconstruction site with better sensibility.

^b^ Table 3 presents NAC sensation in detail.

* *p* < 0.05.

** *p* < 0.01.

As Table 2 points out, temperature sensation did not differ significantly between the two procedures (*p* > 0.3). Also, the evaluation of the pinch tip sensation above the NAC using the Semmes-Weinstein monofilaments was equal in the two groups with 55.6% of the patients in each group feeling the monofilaments sized 4.0–0.07 g. Concerning sensation of skin areal below the NAC (50.0% vs. 36.1%), the ABR group was slightly superior to the IBR group without reaching statistical significance. As shown in Table 3, 45.5% (IBR) and 40% (ABR) patients reported, respectively, high NAC sensation as defined by the thinnest threshold of 0.07 g in the NAC of their nonoperated breast, whereas the highest proportion of patients felt no sensation or just the thickest threshold of 300 g (ABR: 66.7%; IBR: 65.1; n.s.) in the NAC of their nipple-sparing mastectomy. In both categories, no major differences between the two procedures could be shown (*p* > 0.05). Sensation in the reconstructed NACs was not significantly inferior to the preserved NACs, with the IBR patients showing slightly better sensation compared to the ABR patients with 29.6% still feeling a threshold of 2.0 g (n.s.).

**Table 3:** Evaluation of NAC sensitivity.

Items (mean, SD)	IBR	ABR	*p*-value
NAC not-operated, *N* (%)			
Semmes-Weinstein	*N* = 44	*N* = 25	0.613
No	3 (6.8)	1 (4.0)	
300 g	2 (4.5)	1 (4.0)	
4.0 g	0	2 (8.0)	
2.0 g	15 (34.1)	5 (20.0)	
0.4 g	4 (9.0)	4 (16.0)	
0.07 g	20 (45.5)	12 (48.0)	
Temperature	33 (75.0)	17 (68.0)	0.116
NAC preserved, *N* (%)			
Semmes-Weinstein	*N* = 63	*N* = 21	0.504
No	31 (49.2)	9 (42.9)	
300 g	10 (15.9)	5 (23.8)	
4.0 g	12 (19.0)	2 (9.5)	
2.0 g	8 (12.7)	2 (9.5)	
0.4 g	3 (4.8)	3 (14.3)	
0.07 g	0	0	
Temperature	12 (19.0)	7 (33.3)	0.322
NAC reconstructed, *N* (%)			
Semmes-Weinstein	*N* = 27	*N* = 20	0.348
No	10 (37.0)	9 (45.0)	
300 g	6 (22.2)	6 (30.0)	
4.0 g	3 (11.1)	4 (20.0)	
2.0 g	8 (29.6)	1 (5.0)	
0.4 g	0	0	
0.07 g	0	0	
Temperature	7 (25.9)	0	0.318

NAC, Nipple-areola complex; SD, standard deviation.

The aesthetic outcome was evaluated separately depending on the laterality (unilateral/bilateral reconstruction), Table 4 and Figures 1A–D. In almost every category in both groups, an average of 3 (neutral) to 4 (satisfied) point scores were achieved. Only the scars in the unilateral ABR patient group (2.88 ± 0.71) and the abdominal scar in the bilateral ABR group (2.98 ± 0.87) were ranked less than 3 points. In patients with unilateral reconstruction, ABR tended to score higher in every category (shape, volume, symmetry, NAC size, NAC position, position of the inframammary fold, breast–body relation, and overall breast appearance) without reaching statistical significance despite the evaluation of the scars. In this category, IBR scored significantly higher (*p* = 0.005). The overall breast appearance in both groups was rated significantly higher (*p* = 0.07) in the photographs of the whole upper body compared to the photographs from above the waistline. In patients with bilateral reconstruction, IBR scored higher in every category compared to ABR with only the differences in the evaluation of the scars reaching statistical significance (*p* < 0.001). Again, overall breast appearance was rated significantly higher in the IBR group compared to the photographs that were blinded with regard to the procedure. This was in contrast to the bilateral ABR group with the appearance of the abdominal scar in the photographs impacting negatively the overall breast appearance (3.26 ± 0.85 vs. 3.22 ± 0.97; n.s.).

**Table 4:** Aesthetic outcome.

Items (mean, SD)	IBR	ABR	*p*-value
Unilateral	*N* = 44	*N* = 25	
Blinded			
Shape	3.17 ± 0.85	3.30 ± 0.79	0.547
Volume	3.52 ± 0.73	3.84 ± 0.58	0.075
Symmetry	3.15 ± 1.06	3.38 ± 0.75	0.322
NAC size	3.21 ± 0.89	3.25 ± 0.67	0.847
NAC position	3.30 ± 0.83	3.56 ± 0.69	0.207
Position inframammary fold	3.53 ± 0.61	3.68 ± 0.57	0.333
Scars	3.52 ± 0.94	2.88 ± 0.71	0.005**
Breast–body relation	3.50 ± 0.84	3.64 ± 0.74	0.493
Overall breast appearance	3.29 ± 0.78	3.36 ± 0.69	0.724
Whole body			
Abdominal scar	—	3.32 ± 0.74	—
Overall breast appearance	3.37 ± 0.92	3.45 ± 0.72	0.724
Bilateral	*N* = 28	*N* = 11	
Blinded			
Shape	3.51 ± 1.05	3.22 ± 0.86	0.445
Volume	3.82 ± 0.88	3.80 ± 0.61	0.944
Symmetric	3.77 ± 0.92	3.44 ± 0.64	0.304
NAC size	3.65 ± 0.92	3.06 ± 0.81	0.134
NAC position	3.64 ± 0.92	3.52 ± 0.70	0.724
Position inframammary fold	3.76 ± 0.73	3.42 ± 0.87	0.240
Scars	3.79 ± 1.02	3.52 ± 0.70	0.001**
Breast–body relation	3.86 ± 0.86	3.70 ± 0.71	0.592
Overall breast appearance	3.65 ± 0.97	3.26 ± 0.85	0.268
Whole body			
Abdominal scar	—	2.98 ± 0.87	—
Overall breast appearance	3.89 ± 0.99	3.22 ± 0.97	0.073

NAC, Nipple-areola complex; SD, standard deviation.

* *p* < 0.05.

** *p* < 0.01.

Both the EORTC QLQ-C30 and BR23 questionnaires did not show any significant differences in the outcome scores of the two groups (Table 5). Patients after IBR displayed an improved body image (71.9 ± 29.2 vs. 62.0 ± 34.9; *p* = 0.135) whereas ABR patients showed a superior sexual enjoyment (74.6 ± 23.3 vs. 65.8 ± 26.7; *p* = 0.209). The Breast-Q proofed ABR patients to be significantly more satisfied with the breast (s) than IBR patients (*p* < 0.033), whereas neither statistically significant nor clinically relevant differences were shown in the other subscales.

**Table 5:** EORTC QLQ-C30 and QLQ-BR23 scores in all patients undergoing skin- or nipple-sparing mastectomy.

Items (mean ± SD)	IBR (*n* = 72)	ABR (*n* = 36)	*p*-value
EORTC QLQ-C30			
Global health status/QoL	68.9 ± 20.7	71.8 ± 19.9	0.502
Functional scales			
Physical functioning	85.6 ± 17.6	81.7 ± 19.4	0.327
Role functioning	76.0 ± 28.5	75.5 ± 28.2	0.930
Emotional functioning	62.4 ± 29.9	64.4 ± 28.8	0.753
Cognitive functioning	75.1 ± 28.6	77.4 ± 7.7	0.698
Social functioning	74.4 ± 29.6	70.7 ± 32.3	0.560
EORTC QLQ—BR 23			
Functional scales, *N* (%)			
Body Image	71.9 ± 29.2	62.0 ± 34.9	0.135
Sexual functioning	38.1 ± 24.8	35.9 ± 30.4	0.691
Sexual enjoyment	65.8 ± 26.7	74.6 ± 23.3	0.209
Future perspective	50.2 ± 33.6	57.3 ± 36.2	0.341
Breast-Q			
Physical wellbeing			
Sexual	58.6 ± 21.9	59.5 ± 21.3	0.864
Chest	67.6 ± 13.9	71.4 ± 15.9	0.214
Abdomen	—	68.8 ± 25.9	—
Psychosocial wellbeing	73.5 ± 20.3	76.7 ± 20.4	0.449
Patient satisfaction with			
Breast	59.9 ± 17.9	67.7 ± 15.6	0.033*
Nipple (*n* = 23)	—	59.3 ± 28.6	—
Outcome	73.2 ± 20.2	78.4 ± 19.0	0.201
CES-D			
Mean (SD)	10.2 ± 8.9	9.3 ± 7.7	0.589
Score ≥ 18, *N* (%)	10 (14.3%)	7 (20%)	0.575

QoL, quality of life; CES-D, Center for Epidemiology Studies Depression Scale; SD, standard deviation.

* *p* < 0.05.

** *p* < 0.01.

Furthermore, CES-D scores were comparable in the two study groups (IBR: 10.2 ± 8.9; ABR: 9.3 ± 7.7) with 10 IBR patients (14.3%) and 7 ABR patients (20%) scoring 18 points or higher, which indicates a major depression.

## Discussion

Previous studies comparing ABR to IBR concentrated mostly on “patient-reported satisfaction with breast” using the Breast-Q (8, 10, 11, 17). Only a few studies compared the aesthetic outcome (18–20) as well as the quality of breast and NAC sensitivity (3, 21, 22) with the majority of them displaying a small patient collective (23). We could show that IBR is associated with a higher percentage of major complications, while ABR is very often followed by secondary procedures to improve the aesthetic outcome. In unilateral reconstruction with ABR, more aesthetically pleasing results were achieved, while in bilateral reconstruction, IBR was superior. Despite the poorer scar appearance, ABR patients were more satisfied with the breast and overall outcome.

We could show that IBR patients suffered more often from major complication (30.6% vs. 8.3%) including major CC (25%), implant dislocation (8.3%), or major infection (1.4%) leading to an implant exchange once, twice, or more often (30.6%). CC rates are in line with the current literature suggesting varying rates of CC after IBR between 10% and 45% (24, 25) depending on irradiation of the breast, type of implant, and surgical technique (25). Since we had a significant difference concerning the time-span of follow-up (IBR: 27.1 ± 9.3 vs. ABR: 34.9 ± 20.5), we must assume that rates of CC with the need of implant exchange in our study cohort are going to increase even more in the future (26), whereas in ABR, no further major complication have to be expected (26).

In all of our ABR patients with PMRT, ABR was performed secondarily, which is in line with former recommendations (27). In contrast, recent literature studies show acceptable complication rates also in ABR when performed immediately, hence before PMRT (28, 29). Therefore, ABR in patients with PMRT, regardless of the timing, is highly recommended in this patient cohort (28, 30). In total, 20.9% of IBR patients had a history of PMRT, 15.5% of them after reconstruction. Both PMRT before (with/without immediate tissue expander reconstruction) and after IBR-reconstruction have been associated with major CC and implant removal (25, 31). This finding can further explain counts of major complications in our IBR patient cohort and underlines the necessity of a detailed informed consent including the risks of IBR after PMRT.

The average BMI of the ABR patients was 26.9 kg/m^2^ indicating overweight compared to the normal-weighed IBR patients (24.3 kg/m^2^). Overweight is associated with higher total complication rates (32), which might explain the higher rates of minor complications such as haematoma and minor wound healing disorders of the breast compared to IBR. Anyway, ABR patients stayed significantly longer in the hospital. However, this can rather be explained by the necessity for close perfusion monitoring of the microsurgical procedure with subsequent mobilisation.

In contrast to the rates of major complications, ABR patients required secondary procedures significantly more often to improve breast shape and scar appearance at the breast and donor site or contralateral reduction mammaplasty. The reason might be the more challenging shaping of the new breast with the existing autologous tissue as well as the need for two surgical sites (breast and donor site) (8). Nonetheless, these secondary procedures were not medically indicated and represent patients’ high priority of the aesthetic outcome of breast reconstruction. This leads to the assumption of a high correlation of aesthetic outcome and QoL.

Several previous studies evaluating aesthetic outcome after breast reconstruction reported superior results in ABR patients, but did not differentiate between uni- and bilateral reconstruction when comparing ABR and IBR (7, 19). Interestingly enough, we found ABR to be more aesthetically pleasing in unilateral reconstructions, whereas in bilateral reconstruction, results of IBR were superior. Cohen et al. stated comparable surgical results with regard to symmetry in both ABR and IBR in unilateral reconstruction (18). Again, the more challenging and additionally symmetrical shaping of the new breasts with autologous tissue compared to the use of two identical implant prostheses might be the reason for the superiority of IBR in bilateral reconstruction. Furthermore, in unilateral abdominal-based ABR, both sides of donor tissue can be used, whereas in bilateral ABR, the use of the same amount of tissue might result in small breasts. Anyway, in line with the existing literature, IBR showed a superior scar appearance (19), which also corresponds to the more frequent scar revisions performed after ABR.

Consistent with previous findings, the Breast-Q showed the ABR patients to be more satisfied with the breast and overall outcome and to have a superior psychosocial and physical wellbeing (chest); however, only the differences in “satisfaction with breast” reached statistical significance (8, 10, 17). Statistical significance might have been achieved in the other subscales with a bigger sample size in the ABR group. Nevertheless, mean scores were comparable to the numbers stated by Misere et al. (Breast: ABR 68.3, IBR 55.5; Outcome: ABR: 70.9, IBR: 60.0) and Weichman et al. (Breast: ABR 73.8, IBR 63.7; Outcome: 76.0, IBR 73.1) (10, 17). In contrast, concerning the health-related QoL (EORTC QLQ-C30 and BR23), none of the procedure showed to be superior.

Even though there was no significant difference in CES-D results between the two groups, the fact that 14.3% of IBR patients and a fifth of ABR patients scored at least 18, which indicates a major depression, is worrying. While psycho-oncological support has become an inherent part of cancer treatment, our results indicate that even with 27 months (IBR) and 35 (ABR) months, respectively, of follow-up since breast reconstruction, the availability of psychological help if needed remains of some importance.

Furthermore, there were no major differences between the two procedures concerning sexual wellbeing (Breast-Q) and sexual functioning (EORTC QLQ-C30), while sexual enjoyment (EORTC QLQ-C30) tended to be superior in the ABR group.

Besides regaining the “female”-body image and, therefore, an aesthetically pleasing outcome, we assumed sexuality to be another central aspect in breast reconstruction. Therefore, we evaluated breast and NAC sensitivity. According to the findings by Santanelli et al in both groups, breast and nipple sensation was best above the NAC whereas the majority of patients had no pinch tip sensation at the NAC or below no matter what group they belonged to (33). Nevertheless, breast and nipple sensation were significantly impaired, which is in line with previous studies stating a significantly reduced tactile, thermal, and nociceptive cutaneous sensitivity after both mastectomies with spared and regrafted NACs (3, 21). Interestingly enough, we could not find a superiority of NAC sensation in nipple-sparing mastectomies (NAC preserved) compared to reconstructed NAC which is in line with the findings of Gahm et al. (3). Since there were no major differences in breast and NAC sensitivity between the two procedures, this does not serve to be an explanation for the superior sexual enjoyment in ABR.

### Limitations

Limitations of the study include the cross-sectional design and the small sample size above all in the ABR group with a possible selection bias in terms of “too good” or “too bad” results. Reasons for study denial may include satisfactory results and, therefore, no further interest in another outpatient appointment as well as a major psychological burden associated with the breast reconstruction. Accordingly, patients might have accepted to combine study participation with a presentation in the outpatient department to talk about existing problems or, on the contrary, were so thankful that they wished to share their experiences with future patients. Furthermore, we did not provide long-term follow-up data, which limit the ability to finally evaluate CC rates.

## Conclusion

We conclude that both procedures achieve sufficient results in terms of patient satisfaction, HRQoL, and aesthetics. IBR patients must expect higher rates of major complications like CC with the need of a future implant exchange, whereas ABR patients more often require secondary procedures to improve the aesthetic result. Since one-fifth of IBR patients had a history of PMRT, which is highly associated with higher complication rates, general (contra-) indications should be considered in the decision-making process. In unilateral reconstruction, ABR achieved a superior aesthetic result, while in bilateral reconstruction, IBR was aesthetically more pleasing. Anyway, ABR patients were more satisfied with the breast (s) despite a more severe scar appearance both at the breast and the donor site. Regardless of the procedure and whether NAC was preserved or reconstructed, patients have to accept a severely impaired breast and NAC sensation. Whether this compromises sexual functioning and enjoyment has to be further explored. Also, this outlines the importance of outcome analysis of neurotisation procedures.

Since we should comprehend each patient as an individual with different expectations and perceptions not to mention physical characteristics, we have to distance from the “one-size-fits all” assumption meaning that neither ABR nor IBR serve as the superior technique for every patient. The results of our study may contribute to help surgeons to give an overview of the advantages and disadvantages of each procedure and, therefore, involve patients into their individual decision-making process.

## Data Availability

The raw data supporting the conclusions of this article will be made available by the authors, without undue reservation.
